# Circular RNA circFOXO3 regulates KDM2A by targeting miR‐214 to promote tumor growth and metastasis in oral squamous cell carcinoma

**DOI:** 10.1111/jcmm.16533

**Published:** 2021-06-12

**Authors:** Yilong Ai, Siyuan Wu, Chen Zou, Haigang Wei

**Affiliations:** ^1^ School of Stomatology and Medicine Foshan Stomatology Hospital Foshan University Foshan China

**Keywords:** circFOXO3, KDM2A, miR‐214, OSCC

## Abstract

Oral squamous cell carcinoma (OSCC) is a pathological type of oral cancer, which accounts for over 90% of oral cancers. It has been widely shown that circRNA is involved in the regulation of multiple malignant oral diseases including OSCC. However, the mechanism underlying how circRNA regulates OSCC is still not clearly elucidated. In this article, we report circFOXO3 promotes tumor growth and invasion of OSCC by targeting miR‐214 which specifically degrades the lysine demethylase 2A (KDM2A). CircRNA sequencing was conducted in OSCC tumor and tumor‐side tissues, and the expression of circFOXO3 is found to be markedly increased in tumor tissues. CircFOXO3 is also highly expressed in several OSCC cell lines compared with human oral keratinocytes. Transwell assay and colony formation showed that knockdown of circFOXO3 prevents the invasion and proliferation of oral cancer cells. Via bioinformatic research, miR‐214 was found to be the target of circFOXO3 and correlate well with circFOXO3 both in vitro and in vivo. KDM2A was then validated by database analysis and luciferase assay to be the direct target of miR‐214. KDM2A helps to promote tumor invasiveness and proliferation of OSCC. Collectively, our results proved that circFOXO3 sponges miR‐214 to up‐regulate the expression of KDM2A, thus promotes tumor progression in OSCC.

## 1 INTRODUCTION

Oral cancer represents the eighth most common cancer and a major health problem worldwide with an estimated annual incidence of more than 500 000 cases.^1^ Oral squamous cell carcinoma (OSCC) is a pathological type of oral cancer, which accounts for over 90% of oral cancers.^2^, ^3^ Even though there have been advances in screening and treatment of oral cancer in recent years, OSCC patients are often diagnosed at late stages, which results in missing the best window period for treatment.^2^, ^3^ Thus, it is meaningful to investigate the molecular mechanisms underlying OSCC pathogenesis and establish efficient markers for early diagnosis of this disease.

MicroRNAs (miRNAs) were found to play crucial roles in major cancers including OSCC. In addition, RNA sequencing (RNAseq) has identified multiple families of non‐coding RNAs, such as long intergenic non‐coding RNAs (lncRNA) and circular RNAs (circRNAs).^4^, ^5^, ^6^ Compared with linear RNAs, circRNAs are more stable due to the non‐canonical splicing without a free 3’ or 5’ end.^6^, ^7^ CircRNA can function as miRNA sponges, whose sequences can competitively bind miRNAs to regulate the expression of target genes.^6^ Our investigations focused on circular RNA circFOXO3 (also termed as *Hsa_circ_0006404*), which is markedly increased in OSCC tumor tissues and has been reported to be related to tumor progression.^8^, ^9^

The lysine demethylase KDM2A, also known as JHDM1A or FBXL11, was the first JmjC‐domain containing demethylase to be identified.^10^, ^11^ KDM2A demethylates mono‐ and di‐methylated lysine K36 of histone H3(H3K36me1/2),^12^ thus functions as a transcriptional repressor of targeted gene promoters.^13^, ^14^ KDM2A has recently been shown to promote breast cancer cell proliferation by activating rRNA genes.^15^

In this study, we demonstrated that circFOXO3 up‐regulates KDM2A by targeting miR‐214 to promote OSCC cell invasion and progression. Our results manifested that regulation of KDM2A by circFOXO3‐miR‐214 may take part as a novel therapeutic strategy for OSCC treatment.

## 2 MATERIALS AND METHODS

### 2.1 Cells culture

FaDu, Cal27, UM1, UM2, SCC‐4, SCC‐9, SCC‐15, SCC‐25 and human oral keratinocyte (HOK) and human gingival fibroblasts (HGF) cells were obtained from the Cell Bank of the Chinese Academy of Science (Shanghai, China) and American Type Culture Collection (Manassas, VA, USA). All the cell lines were cultured under the introduction of the manufacturer.

### 2.2 Clinical tissues

Thirty‐six paired OSCC and adjacent normal tissues were obtained from patients who received surgery in Foshan Stomatology Hospital. None of them received chemoradiotherapy prior to surgery. The study protocol was approved by the Ethics Committee of Foshan Stomatological Hospital, School of Stomatology and Medicine, with written informed consent obtained from all patients. The relevant clinical information was provided in Table S1.

### 2.3 Real‐time PCR analysis of mRNA

Total RNA was isolated with TRIzol reagent. The cDNA was synthesized from 2 μg of RNA by the Quantscript RT Kit (TianGen, KR103). The primer sequences of RT‐PCR were as follows: circFOXO3, forward: GTGGGGAACTTCACTGGTGCTAAG, reverse: GGGTTGATGATCCACCAAGAGCTCTT; miR‐214‐5p, forward: TGCCTGTCTACACTTGCTGTGC, reverse: GGTGCAGGGTCCG AGGTAT; KDM2A, forward: CTCCCTTGAGCTTGGTTCTG, reverse: AATCCACTTGGGTAGCAACG; Actin, forward: CCAACCGCG AGAAGATGA, reverse: CCAGAGGCGTA CAGGGATAG; U6, forward: CTCGCTTCGGCAG CACA, reverse: AACGCTTCACGAATTTGCGT.

### 2.4 Western blotting

Western blot was conducted as previously described.^6^ In brief, the cells were harvested with a scraper and then washed once with cold PBS. The cells were then lysed in lysis buffer containing 50 mmol/L Tris‐HCl, 250 mmol/L NaCl, 5 mmol/L EDTA, 50 mmol/L NaF, 0.1% NP‐40 and 1% protease inhibitor cocktail. Equal amounts of proteins were size‐fractionated by 7.5%‐15% SDS‐PAGE. KDM2A antibody (24311‐1‐AP) was purchased from Proteintech; beta‐Actin (sc‐7210) was purchased from Santa Cruz Biotechnology Inc Data collected came from at least three independent experiments.

### 2.5 RNA immunoprecipitation assay

RIP assay was conducted using Magna RIP Kit (EMD Millipore). Cells were lysed in RIP lysis buffer, and the cell lysate was treated with magnetic beads conjugated to human anti‐Ago2 antibody (Millipore) or control antibody (normal mouse IgG; Millipore). qRT‐PCR was performed to detect circFOXO3 expression.

### 2.6 Colony formation assay

Cells were plated and transfected with indicated siRNA/plasmid and then cultured for 48 hours. Cells were trypsinized and plated into 60‐mm plates with the concentration of 5000 cells/plate. After 2 weeks, methanol fixation and staining with methylene blue was undertaken to identify visible colonies. Plating efficiencies were calculated as follows: number of colonies formed/number of cells plated.

### 2.7 Transwell migration assay

A total of 2 × 10^4^ SCC‐4 or SCC‐9 cells with transfection treatments for 48 hours were re‐suspended in DMEM medium (200 μL) and then seeded into the upper chambers of transwell plates (8 μm size, Corning), respectively. After that, 600 μL DMEM medium with 10% FBS was added to the lower chamber. The non‐migrated cells were removed after incubation for 24 hours at 37°C. Cells that migrated to the bottom of the membrane were fixed with paraformaldehyde (4%). After staining with crystal violet (0.1%), cell counting process was carried out with a 200× microscope (Olympus Corporation).

### 2.8 CCK‐8 assay

Equal numbers of cells (approximately 5000/well) were seeded into a 96‐well plate 24 hours before experimentation. Cells were transfected with different plasmid or small RNA and then cultured for indicated time. After treatment, CCK‐8 was added into the 96‐well plate and incubated at 37°C for 1 hour. The absorbance of each sample was read at 450 nm.

### 2.9 Reporter vectors constructs and luciferase reporter assay

The fragments of KDM2A (containing predicted binding sites) were cloned into the pMIR‐REPORT Vector (Promega) to form the reporter vector KDM2A wild type (KDM2A‐wt). The sequence replacing the putative binding site was named KDM2A mutant (KDM2A‐mut). The vector and the miR‐214 were cotransfected into HEK 293T cells to test the luciferase activity by Dual‐Luciferase Reporter Assay System (Promega).

### 2.10 Bioinformatics

We used Circinteractome database to predict circFOXO3 potential miRNA. We applied TargetScan to search miRNA‐targeted genes.

### 2.11 Tumorigenesis in nude mice

Four‐week‐old BALB/c nude mice were purchased from the Experimental Animal Centre of Peking University Health Science Centre and housed in a pathogen‐free environment. DLD1 cells (~1 × 10^7^) were delivered into animals via hypodermic injection. After 2 weeks, all mice were randomly divided into four groups (n = 6) and administered adenovirus via tail intravenous injection. At the end of the experiment (5 weeks after tumor implantation), the mice were sacrificed and the weight of each tumor was determined.

### 2.12 Statistical analysis

For all statistical tests, three or more independent experiments were performed, data are shown as means ± SD *P* < .05, by unpaired Student's *t* test, was considered statistically significant. Data were analyzed by Graphpad version 8.0 (Graph Pad Software).

### 2.13 Study approval

This study was approved by the Ethics Committee of Foshan Stomatology Hospital Affiliated to Foshan University, Foshan, Guangdong, China. Informed consent was obtained from each patient before the use of their tumor tissues.

## 3 RESULTS

### 3.1 CircFOXO3 is significantly increased in oral squamous tumors and OSCC cell lines

**FIGURE 1:**
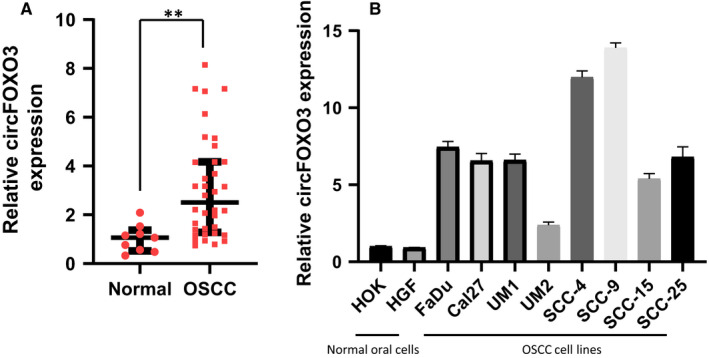
The level of circFOXO3 in OSCC tumors and cell lines. A, Relative expression of circFOXO3 in OSCC tumor (n = 36) and tumor‐side tissues (n = 9). B, Total RNA was collected after incubation of the cells for 24 h. Quantitative PCR (qPCR) was performed to examine the relative expression of circFOXO3 in various OSCC cell lines, normal human oral keratinocytes(HOK) and human gingival fibroblasts(HGF). Data are presented as means ± SD (n = 3). *P < .05, **P < .01 [Colour figure can be viewed at wileyonlinelibrary.com]

In order to explore the potential role of circFOXO3 in oral cancer, we firstly detected the expression of circFOXO3 in oral squamous tumor and tumor‐side tissue samples from OSCC patients. As shown in Figure 1A, the expression of circFOXO3 was significantly increased in tumor tissues compared with tumor‐side tissues. Furthermore, we examined the expression of circFOXO3 in two normal oral cells and eight OSCC cell lines. Relatively higher levels of circFOXO3 were observed in OSCC cell lines compared with normal oral cells (Figure 1B). These results suggested that circFOXO3 may be involved in regulation of oral cancer growth.

### 3.2 CircFOXO3 is required for the invasiveness and proliferation of OSCC

**FIGURE 2:**
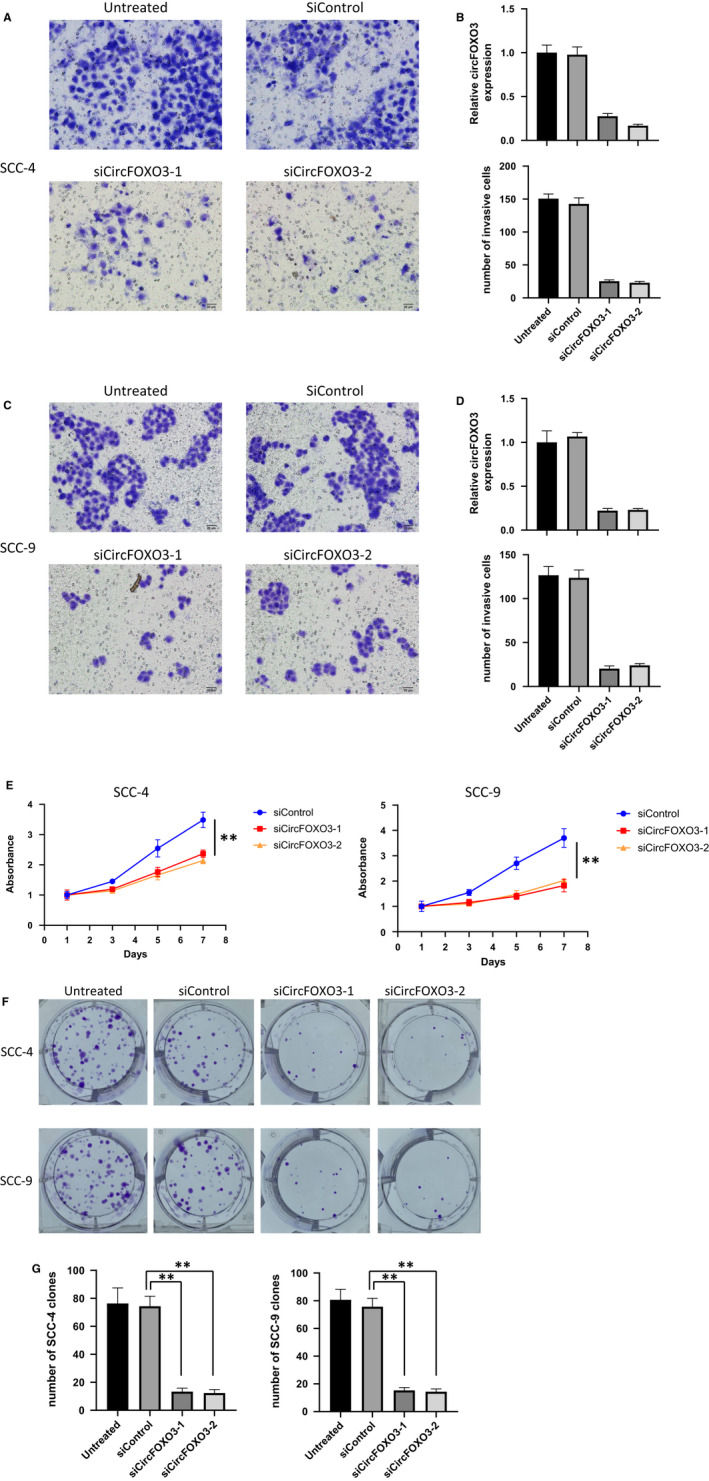
circFOXO3 is required for the invasiveness and proliferation of OSCC. (A,C) The effects of siRNA against circFOXO3 on both SCC‐4 and SCC‐9 cell invasion. A total of 1 × 10^5^ transfected cells were seeded onto a permeable membrane in a Boyden chamber to allow the cells to invade the opposite layer of the membrane. (B,D) Quantitative analysis of circFOXO3 expression and invaded numbers of the cells with indicated treatment. Data are presented as means ± SD (n = 3). **P < .01. E, Effect of circFOXO3 knockdown on the proliferation of SCC‐4 and SCC‐9 cells. Cells were transfected with siControl or si‐CircFOXO3 and cultured for up to seven days. Cell proliferation was measured by CCK‐8 assay. Data are presented as means ± SD (n = 3). **P < .01. F, Proliferation of SCC‐4 and SCC‐9 cells transfected with siControl or siCircFOXO3, as detected by colony formation assay. G, Quantitative analysis of clone numbers with indicated treatment. Data are presented as means ± SD (n = 3). **P < .01

The increased expression of circFOXO3 in OSCC indicated that circFOXO3 may promote tumor growth. To test this hypothesis, we firstly measured the invasive abilities of two OSCC cell lines specifically treated with circFOXO3 knockdown (Figure 2A‐D). As shown in Figure 2A,C, knockdown of circFOXO3 markedly reduced the invasiveness of both SCC‐4 and SCC‐9 cells by transwell invasion assay. For long‐term cell proliferation, CCK‐8 cell growth assay was performed to investigate the effect of circFOXO3. CircFOXO3 knockdown significantly prevented cell proliferation of both SCC‐4 and SCC‐9 (Figure 2E). In accordance with this, colony formation assay also manifested reduced clone numbers upon treatment with circFOXO3 siRNA (Figure 2F‐G). Collectively, these results indicated that circFOXO3 might be essential for the progression and invasion of OSCC malignant tumors.

### 3.3 CircFOXO3 functions as a miRNA sponge for miR‐214 in OSCC

**FIGURE 3:**
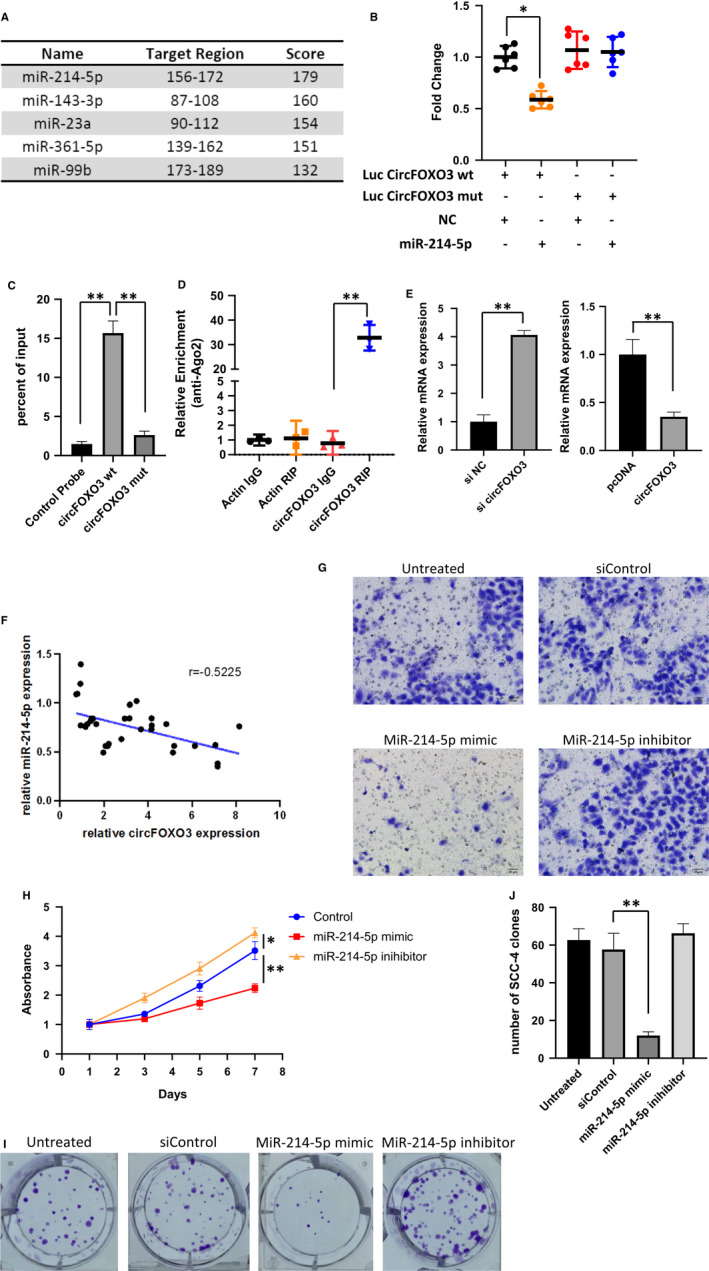
circFOXO3 acts as a miRNA sponge for miR‐214. A, Bioinformatics analysis of circFOXO3 binding miRNAs. B, Luciferase activity was measured in circFOXO3 WT, Mut plasmids with miR‐214 co‐transfected 293T cells. Data are presented as means ± SD (n = 3). *P < .05. C, RNA pull‐down was conducted to measure the miR‐214 expression on circFOXO3 WT and Mut enrichment. Data are presented as means ± SD (n = 3). **P < .01. D, AGO2 IP assay was performed to detect CircFOXO3 levels in 293Tcells stably expressing AGO2. Data are presented as means ± SD (n = 3). **P < .01. E, Relative expression of miR‐214 in si‐NC (Negative Control)/si‐circFOXO3 and NC (Negative Control)/pcDNA‐circFOXO3 infected SCC‐4. Data are presented as means ± SD (n = 3). **P < .01. F, The correlation of circFOXO3 and miR‐214 expression level in OSCC tumor tissues analyzed by Pearson (*r* = .5225, *P* < .05). G, The effects of miR‐214 mimic/inhibitor on both SCC‐4 cell invasion. A total of 1 × 10^5^ transfected cells were seeded onto a permeable membrane in a Boyden chamber to allow the cells to invade the opposite layer of the membrane. H, SCC‐4 cells were transfected as indicated and cultured for different times. Cell viability was measured by CCK‐8 assay. Data represent the means ± SD (n = 3). *P < .05, **P < .01. I, Proliferation of SCC‐4 cells transfected with siControl, miR‐214 mimic or inhibitor, as detected by colony formation assay. J, Quantitative analysis of clone numbers with indicated treatment. Data are presented as means ± SD (n = 3). **P < .01

Previous studies have reported that circRNAs can function as miRNA sponges. By sharing one or more microRNA response elements, circRNAs can bind to miRNAs and in turn arrest miRNA functions. To examine whether circFOXO3 can function as miRNA sponges, bioinformatics software (RegRNA) was used to predict the potential circRNA/miRNA interactions and found circFOXO3 might interact with several miRNAs (Figure 3A). We selected miR‐214, which was shown to be the top hit, for further analysis. Luciferase assay and RNA pull‐down were used to verify the binding of circFOXO3 to miRNA‐214 (Figure 3B‐C). To further confirm the miRNA sponge function of circFOXO3, RNA immunoprecipitation assay was performed in Flag‐Ago2 transfected cells and we found the specific enrichment of endogenous circFOXO3 (Figure 3D). In addition, relative expression of miR‐214 was up‐regulated with circFOXO3 knockdown, as well as down‐regulated with circFOXO3 overexpression (Figure 3E). Consistently, miR‐214 expression in OSCC tumor tissues well correlated with the expression of circFOXO3 (Figure 3F). These results suggested that circFOXO3 functions as a sponge for miR‐214.

To further investigate the function of miR‐214 in OSCC, we transfected SCC‐4 cells with siControl, miR‐214 mimics or miR‐214 inhibitor. MiR‐214 mimic blocked the invasiveness of SCC‐4 while its inhibitor slightly promoted tumor invasive ability (Figure 3G). The long‐term proliferation assay measured by CCK‐8 confirmed the tumor suppressing effect of miR‐214 (Figure 3H). Moreover, colony formation analysis also showed consistent result with the proliferation (Figure 3I,J).

In conclusion, these results demonstrated that circFOXO3 act as a sponge for miR‐214 and promote OSCC tumor progression and invasion by targeting miR‐214.

### 3.4 CircFOXO3 up‐regulates KDM2A expression by targeting miR‐214

**FIGURE 4:**
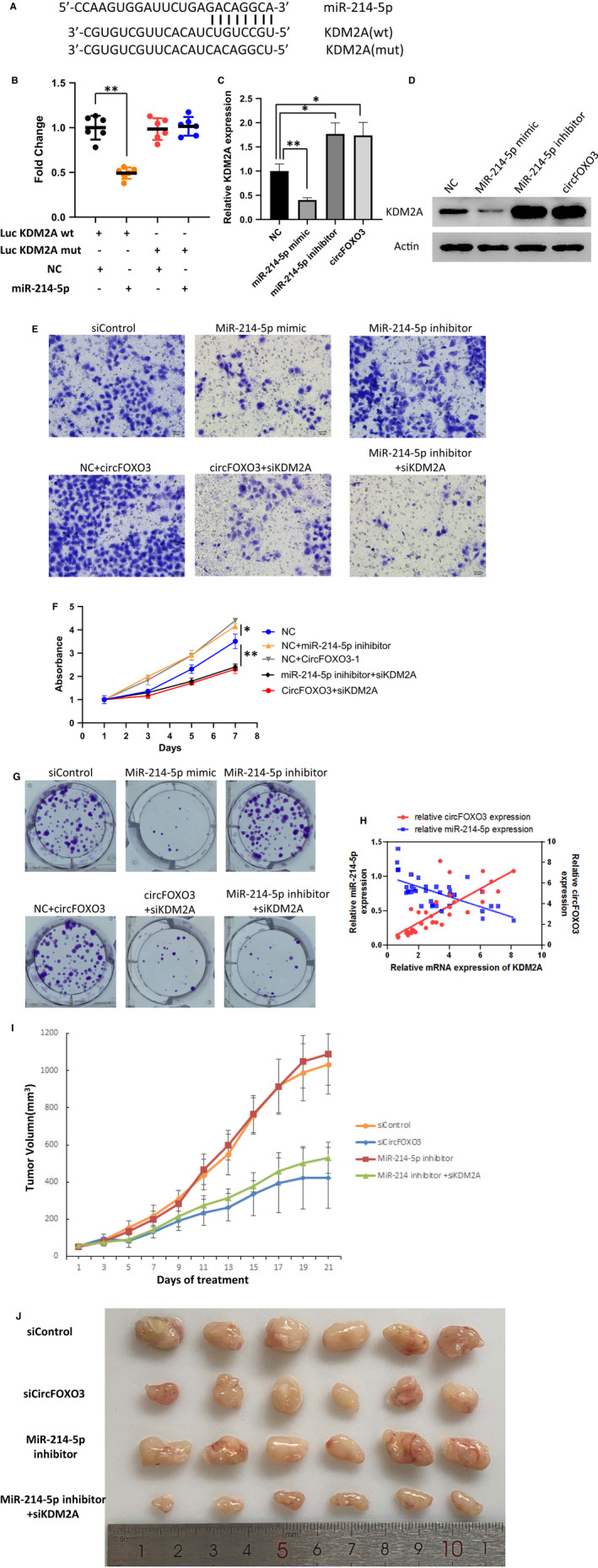
circFOXO3 up‐regulates KDM2A expression by targeting miR‐214. A, Binding sites between miR‐214 and KDM2A was shown. B, Luciferase activity was measured in miR‐214 and KDM2A WT, Mut cDNA plasmids infected 293T. Data are presented as means ± SD (n = 3). **P < .01. C, Relative expression of KDM2A in SCC‐4 cells with indicated treatment. Data are presented as means ± SD (n = 3). *P < .05, **P < .01. D, KDM2A protein level was measured by western blot of SCC‐4 with indicated treatment. E, The effects of KDM2A on both SCC‐4 cell invasion. A total of 1 × 10^5^ transfected cells were seeded onto a permeable membrane in a Boyden chamber to allow the cells to invade the opposite layer of the membrane. F, SCC‐4 cells were transfected as indicated and cultured for different times. Cell viability was measured by CCK‐8 assay. Data represent the means ± SD (n = 3). *P < .05, **P < .01. G, Proliferation of SCC‐4 cells transfected with indicated siRNA/plasmid, as detected by colony formation assay. H, The correlation of miR‐214/KDM2A, circFOXO3/KDM2A expression level in OSCC tumor tissues analyzed by Pearson. I, Four‐week‐old nude mice were engrafted with 1 × 10^7^ SCC‐4 cells and randomly divided into four groups (n = 6). After two weeks, the tumor‐bearing mice were treated with adenovirus expressing different siRNA or inhibitor as indicated. Tumor volumes were calculated by measuring the length and width using Vernier calipers every two days. Data represent the means ± SD (n = 6). J, Images of the tumors for Figure 4I

To further explore the potential target of miR‐214, we searched for the putative gene target of miR‐214 via bioinformatic analysis. KDM2A (Lysine Demethylase 2A) was suggested be a direct target of miR‐214 (Figure 4A). To validate the direct targeting of KDM2A by miR‐214, the wild‐type (wt) KDM2A‐targeted sequence or a mutant variant was cloned into a dual luciferase reporter vector. The effect of miR‐214 on luciferase activity was detected in 293T cells. As shown in Figure 4B, miR‐214 markedly inhibited luciferase activity of wild‐type KDM2A (KDM2A wt), while mutation of the miR‐214‐binding sites (KDM2A mut) abolished the inhibitory effect of miR‐214 (Figure 4B). Tranfection of miR‐214 mimic/inhibitor suggested the consistent effect in regulating KDM2A mRNA expression (Figure 4C). CircFOXO3 showed similar effect as miR‐214 in up‐regulation of KDM2A expression (Figure 4C). And the protein level of KDM2A manifested consistent effect of miR‐214 and circFOXO3 (Figure 4D). Together, these data indicated KDM2A to be a direct target of miR‐214.

To determine the function of KDM2A in circFOXO3‐miR‐214 mediated tumor growth, we co‐infected SCC‐4 cells with KDM2A siRNA and circFOXO3/miR‐214 inhibitor, and detected the invasiveness of SCC‐4 by transwell assay (Figure 4E). Knockdown of KDM2A significantly prevented SCC‐4 invasion in circFOXO3 or miR‐214 inhibitor expression (Figure 4E). CCK‐8 and colony formation assay also manifested the circFOXO3‐miR‐214‐KDM2A axis in regulating OSCC tumor cell growth (Figure 4F‐G). Furthermore, we analyzed the relative expression of miR‐214/KDM2A and circFOXO3/KDM2A in OSCC tumor tissues and found the consistent correlation of both by Pearson (Figure 4H).

To determine whether the effect of circFOXO3‐miR‐214‐KDM2A pathway in vivo, nude mice were injected with SCC‐4 cells and injected with different adenovirus. The tumor sizes were significantly lower in the circFOXO3 knockdown group (Figure 4I,J). In conclusion, these results suggested that circFOXO3 up‐regulates KDM2A expression by targeting miR‐214 thus promotes OSCC invasion and progression.

## 4 DISCUSSION

Oral squamous cell carcinoma is an aggressive malignant tumor with high rates of recurrence and metastasis. The five‐year survival rate of OSCC is only around 20% at late stages and 80% in early stages.^16^, ^17^, ^18^ Deep understanding of OSCC pathogenesis may help to provide more therapeutic target or diagnostic markers for the disease. This study identified the OSCC regulatory ‘CircFOXO3‐miR‐214‐KDM2A’ axis and may provide a novel target for OSCC therapy.

It has been reported that circRNAs, as newly discovered non‐coding RNAs, are involved in the pathogenesis of OSCC.^2^ Multiple circRNAs, such as circUHRF1, circPVT1, and circ_100290, were described to function as oncogenes to regulate OSCC tumorgenesis and progression.^19^, ^20^, ^21^ CircFOXO3 has been reported to be down‐regulated in breast cancer and non‐small cell lung cancer to act as a powerful tumor suppressor by sponging specific miRNAs by targeting the parental transcript FOXO3.^22^, ^23^, ^24^ However, the role of circFOXO3 in OSCC has not been reported. Our findings indicate opposite function of circFOXO3 in regulating tumor growth and invasion. We speculate that the dichotomous effect of circFOXO3 may arise with different signaling pathways in specific tumors.

It has been described that KDM2A is frequently overexpressed in non‐small cell lung cancer tumors and gastric cancers and can promote the growth and motility of cancer cells.^25^, ^26^ This study is the first to report that KDM2A is important for OSCC tumor progression and invasion. Therefore, KDM2A may be a new therapeutic target for oral cancer treatment. Our future study will investigate the mechanism in which KDM2A regulates OSCC cell invasion.

Taken together, we have identified the CircFOXO3‐miR‐214‐KDM2A axis as a novel target for the prevention and treatment of OSCC. The overexpression of circFOXO3 in OSCC provides new promising clinical biomarker for oral cancer diagnose. Besides, regulation of miR‐214 and KDM2A may also serve as an effective therapeutic target for OSCC treatment.

## CONFLICT OF INTEREST

The authors have declared that no competing interest exists.

## AUTHOR CONTRIBUTIONS

**Haigang Wei:** Conceptualization (equal); Supervision (equal); Writing‐original draft (equal); Writing‐review & editing (equal). **Yilong Ai:** Formal analysis (equal); Investigation (equal); Methodology (equal); Resources (equal). **Siyuan Wu:** Data curation (equal); Investigation (equal); Methodology (equal); Resources (equal); Software (equal). **Chen Zou:** Formal analysis (equal); Supervision (equal); Writing‐original draft (equal).

## Supporting information

Table S1

## Data Availability

All data generated or analyzed during this study are included in this article. Further details are available on request.
